# Durable Effect of Acupuncture for Chronic Neck Pain: A Systematic Review and Meta-Analysis

**DOI:** 10.1007/s11916-024-01267-x

**Published:** 2024-06-10

**Authors:** Jiufei Fang, Hangyu Shi, Weiming Wang, He Chen, Min Yang, Shuai Gao, Hao Yao, Lili Zhu, Yan Yan, Zhishun Liu

**Affiliations:** 1 Department of Acupuncture, Guang’anmen Hospital, China Academy of Chinese Medical Sciences, No.5 Beixiange St, Xicheng District, Beijing, 100053 China; 2 Graduate College, Beijing University of Chinese Medicine, Beijing, China

**Keywords:** Durable effect, Acupuncture, Chronic neck pain, Systematic review, Meta-analysis

## Abstract

**Objective:**

Chronic neck pain, a prevalent health concern characterized by frequent recurrence, requires exploration of treatment modalities that provide sustained relief. This systematic review and meta-analysis aimed to evaluate the durable effects of acupuncture on chronic neck pain.

**Methods:**

We conducted a literature search up to March 2024 in six databases, including PubMed, Embase, and the Cochrane Library, encompassing both English and Chinese language publications. The main focus of evaluation included pain severity, functional disability, and quality of life, assessed at least 3 months post-acupuncture treatment. The risk of bias assessment was conducted using the Cochrane Risk of Bias 2.0 tool, and meta-analyses were performed where applicable.

**Results:**

Eighteen randomized controlled trials were included in the analysis. Acupuncture as an adjunct therapy could provide sustained pain relief at three (SMD: − 0.79; 95% CI − 1.13 to − 0.46; p < 0.01) and six (MD: − 18.13; 95% CI − 30.18 to − 6.07; p < 0.01) months post-treatment. Compared to sham acupuncture, acupuncture did not show a statistically significant difference in pain alleviation (MD: − 0.12; 95% CI − 0.06 to 0.36; p = 0.63). However, it significantly improved functional outcomes as evidenced by Northwick Park Neck Pain Questionnaire scores 3 months post-treatment (MD: − 6.06; 95% CI − 8.20 to − 3.92; p < 0.01). Although nine studies reported an 8.5%–13.8% probability of adverse events, these were mild and transitory adverse events.

**Conclusion:**

Acupuncture as an adjunct therapy may provide post-treatment pain relief lasting at least 3 months for patients with chronic neck pain, although it is not superior to sham acupuncture, shows sustained efficacy in improving functional impairment for over 3 months, with a good safety profile.

**Supplementary Information:**

The online version contains supplementary material available at 10.1007/s11916-024-01267-x .

## Introduction

Neck pain, defined as pain extending from the upper cervical line to the level of the scapulae, may manifest as radiating discomfort affecting the head, trunk, and upper limbs [1]. This condition is the second leading cause of disability globally [2••], with an estimated 67% of individuals experiencing neck pain at some point in their lives [3]. Around 20% of these cases progress to chronic neck pain (CNP) [4], a pain persisting for over 3 months [5]. Individuals with sedentary occupations are particularly prone to CNP due to prolonged static postures, inadequate physical activity, and various workplace-related factors. CNP also frequently arises from traumatic events [6]. Notably, CNP imposes a significant public health and economic burden, with about 25% to 60% of patients experiencing pain for a year or longer after the initial episode [7•], a figure that rises to 60–80% among manual laborers [8].

Clinical guidelines typically advocate for oral medications and various interventional strategies for managing CNP. Despite the emphasis on physical therapy, adherence to prescribed exercises is often challenging, resulting in suboptimal outcomes. While physical therapy may offer temporary pain relief, the recurring nature of CNP necessitates frequent clinic visits for continued management, underscoring the limitations of current interventional strategies in providing long-term relief [9]. The conventional first-line treatment for CNP typically recommends the use of nonsteroidal anti-inflammatory drugs (NSAIDs). However, their use is marred by significant safety concerns, leading to the hospitalization of over 100,000 patients annually due to gastrointestinal complications associated with NSAID usage, as well as approximately 16,500 fatalities each year attributed to these adverse effects in the United States [10]. Given these concerns and the lack of substantial evidence for the sustained effectiveness of both pharmacological and physical interventions, there is a pressing need to explore treatment strategies for CNP that are more effective and safer with better sustained effects.

Many patients are dissatisfied with conventional treatments and seek complementary and alternative medicines (CAM). Acupuncture is a traditional Chinese therapeutic method that achieves healing through needle insertion, with a history of use exceeding 3,000 years in East Asia. It is characterized by minimal side effects and lasting efficacy, and has gained worldwide popularity, with over 14.01 million Americans having received acupuncture treatments [11, 12]. Acupuncture has demonstrated sustained effects in treating various diseases, suggesting that its enduring therapeutic efficacy may be advantageous in alleviating CNP [13, 14]. Current reviews have either overlooked the long-term effects of acupuncture in treating CNP or merely focused on its sustained benefits on pain relief, neglecting to comprehensively evaluate its post-treatment effects on functional improvement and quality of life enhancement [15–17]. Given the propensity of CNP to recur and persist, therapies exhibiting more pronounced sustained effects are worthy of patient recommendation. This meta-analysis aims to systematically assess the sustained effects of acupuncture on CNP, focusing on outcomes of pain, function, and quality of life, and safety assessment [18].

## Methods

This systematic review followed the Preferred Reporting Items for Systematic Reviews and Meta-Analyses (PRISMA) guidelines [19], and it was registered on the International Prospective Register of Systematic Reviews (PROSPERO) under ID No. CRD42023403434. [20].

### Search Strategy

A systematic literature search was conducted using PubMed, Embase, Cochrane Library, Chinese Knowledge Infrastructure, Wanfang Database, and the VIP Database for English and Chinese literature from their inception to March 2, 2024. The search terms included chronic neck pain (e.g., cervical pain, cervicodynia, myofascial pain syndrome, trachelodynia, and neck disorder), acupuncture (e.g., dry needling and electroacupuncture), and randomization (e.g., randomized controlled trials and clinical trials). Search strategies were formulated for different databases (supplementary data sheet S1). We manually screened the references of current reviews to obtain more relevant research.

### Inclusion Criteria

Participants: Adults with neck pain of more than 3 months secondary to mechanical neck disorders, myofascial pain syndrome, cervical spondylosis, cervical spine diseases with radiating pain, and myalgia were included in this study.

**Intervention** The intervention group received various types of acupuncture (e.g., electroacupuncture, warm acupuncture, abdominal acupuncture, auricular acupuncture, and dry needling) for neck pain. Studies comparing the combination of acupuncture with other intervention measures to those using the same types of interventions alone were included.

**Comparison** The control group received sham acupuncture, no treatment or active treatments (nonsteroidal anti-inflammatory drugs, exercise, massage, transcutaneous electrical stimulation, etc.).

**Outcomes** This study evaluated the sustained efficacy of acupuncture in treating CNP by analyzing pain intensity, neck dysfunction, and quality of life during follow-up over 3 months post-treatment. Pain intensity was assessed using the Visual Analog Scale (VAS), McGill Pain Questionnaire (MPQ), and Numerical Rating Scale (NRS). Neck dysfunction was assessed using the Neck Disability Index (NDI) and Northwick Park Neck Pain Questionnaire (NPQ). Quality of life was assessed using the 36-item Short Form Health Survey (SF-36).

**Study Design** Randomized Controlled Trials (RCTs) of acupuncture for CNP were included only if they had a follow-up period of at least 3 months post-treatment.

### Exclusion Criteria

Studies involving patients diagnosed with myelopathy were excluded. Whiplash injury was excluded because it may have had a different natural history. Patients with cervical headache/vertigo but without neck pain were omitted from the study. Additionally, studies on acupoint injection, needle-knife therapy, bee venom acupuncture, and studies comparing different acupuncture methods versus Chinese herbal medicine were excluded.

### Study Selection and Data Extraction

Two experienced researchers (JF and HS) independently conducted the study analysis, initially screening titles and abstracts post-deduplication, followed by full-text reviews based on the predefined inclusion and exclusion criteria. Disagreement was addressed via consensus. Data extraction, performed by the two authors using a predesigned table, was overseen by a senior researcher. The data extracted included the first author, intervention and control measures, sample size, duration of disease, follow-up period, outcome measures, and adverse events.

### Assessment of Risk of Bias

Using the Cochrane Risk of Bias 2.0 tool, a revised framework of the Cochrane Collaboration for RCTs, two independent investigators evaluated the bias present in the studies included in the analysis. This tool covers five key domains: the randomization process, deviations from intended interventions, missing outcome data, outcome measurements, and selection of reported results. Assessments were classified as "low," "high," or "having some concerns." Discrepancies were resolved through discussion, with a third investigator (ZL) stepping in for consensus if needed.

### Data Analysis

Statistical analyses were conducted utilizing Review Manager 5.4 (Cochrane Collaboration, Oxford, UK). The mean difference (MD) and 95% CI were calculated for continuous results. Based on the method described by Wan et al. the median and quartile ranges of continuous data were converted into means and standard deviations. The Cochrane QP value and I^2^ statistics were used to examine the heterogeneity in all meta-analyses. When p value < 0.05 or I^2^ > 50%, indicating significant heterogeneity, the results were combined using a random-effects model. Otherwise, a fixed-effects model was used. A p-value < 0.05 was considered statistically significant. Egger’s test was used to assess publication bias (only for results containing ten or more studies).

## Results

**Fig. 1:**
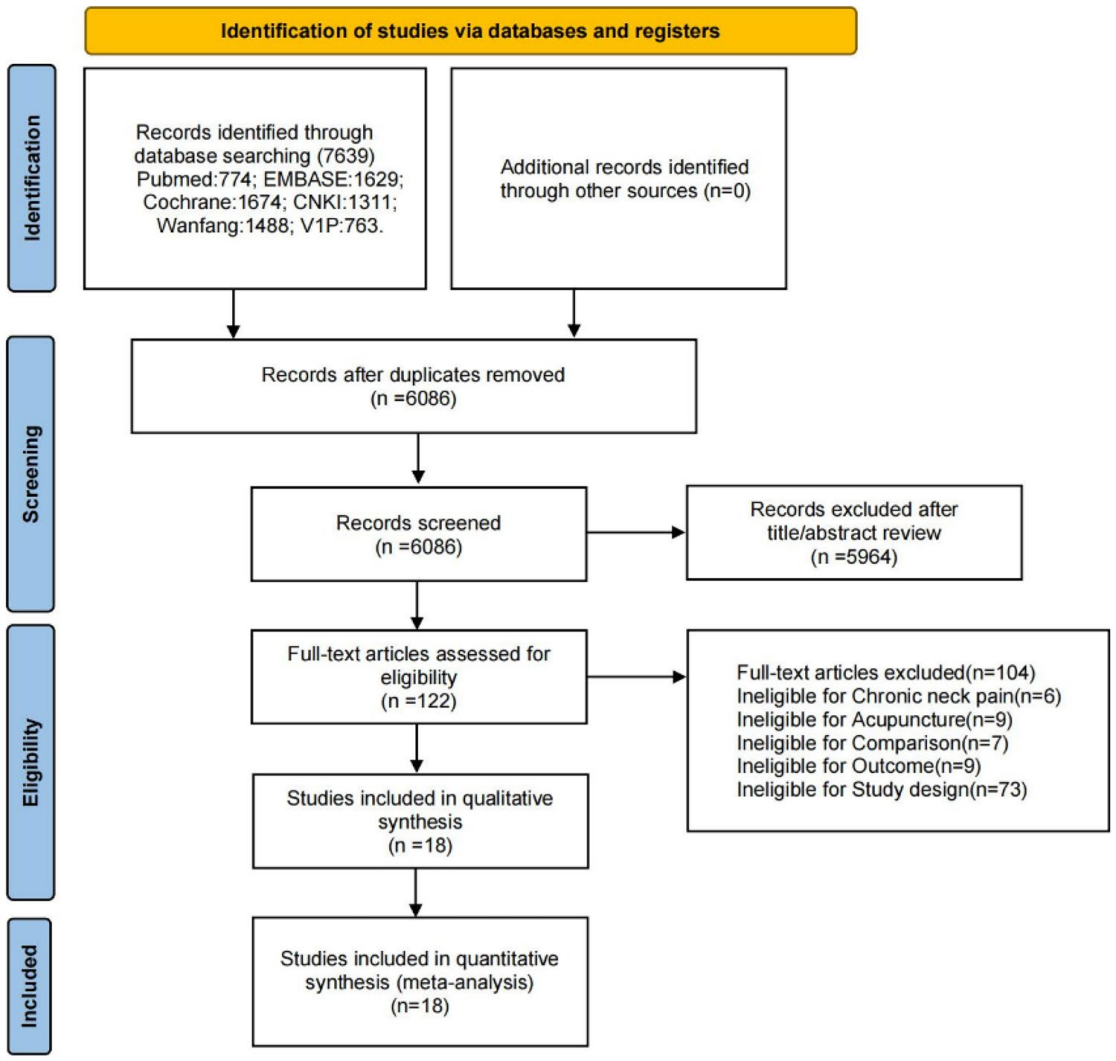
PRISMA flow diagram

The initial search identified 7639 studies from six databases. After excluding 1553 studies due to duplication, 122 studies were screened based on titles and abstracts. Ultimately, 18 articles met the inclusion criteria. The process of study selection is displayed in Fig. 1.

### Characteristics of Included Studies

Eighteen studies [21–38] were included. The sample size, interventions, control, follow-up duration, and outcomes are summarized in Table 1. Interventions included manual acupuncture, dry acupuncture, warm needle moxibustion, and press needles. Seven studies combined acupuncture with manipulation, exercise, or other treatments [25, 26, 29, 32, 36–38]. Control group (CG) can be divided into three categories: sham acupuncture (minimal acupuncture at a non-acupoint or unrelated point, or blunt needle without actually piercing the skin), no treatment, and active treatment (such as TENS, traction treatment, self-exercise, and massage). Pain was assessed using the NRS, VAS, and MPQ; neck dysfunction was assessed using the NDI, NPQ, and NPDS; and quality of life was assessed using SF-36.

**Table 1:** Characteristics of included studies

**Study**	**Diagnosis**	**Intervention**	**Comparison**	**Sample Size**	**Duration of disease**	**Follow-up period**	**Outcomes**	**Adverse events**
**Xu 2014, China** [35]	Cervical spondylosis	Acupuncture	Sham acupuncture	IG:260 CG:266	IG:50.42 ± 65.80 CG:49.94 ± 73.18 (months)	1, 3 months after completion of treatment	NPQ	NR
**Liang 2011, China** [27]	Chronic neck pain	Acupuncture	Sham acupuncture	IG:93 CG:97	≥ 6 months	1, 3 months after completion of treatment	VAS, NPQ, SF-36	8%/6%
**Gattie 2021, USA** [31]	Chronic neck pain	Dry needling + Manual therapy, and exercise	Sham acupuncture + Manual therapy, and exercise	IG:40 CG:37	≥ 3 months	4 weeks, 6 months, 1 year after completion of treatment	VAS, NPQ, NDI	NR
**Witt 2006, Germany** [21]	Chronic neck pain	Acupuncture	No treatment	IG: 1753 CG: 1698	IG:6.0 ± 6.9 CG:6.1 ± 7.3 (years)	3, 6 months after completion of treatment	SF-36	8.9%/NR
**Irnich 2001, Germany** [22]	Chronic neck pain	Acupuncture	Massage	IG:56 CG:60	NR	1 week, 3 months after completion of treatment	VAS	33%/21%
**Ilbuldu 2004, Turkey** [23]	Chronic mechanical neck pain	Dry needling	Laser	IG:20 CG:20	IG:38.48 ± 31.94 CG:36.95 ± 33.65 (months)	3 months after completion of treatment	VAS	NR
**Salter 2006, UK** [24]	Chronic neck pain	Acupuncture	Medication, massage, and recommended exercise	IG:10 CG:14	IG:5.7 ± 6.4 CG:5.5 ± 5.5 (years)	1, 3 months after completion of treatment	NPQ	60%/ NR
**Franca 2008, Brazil** [25]	Chronic mechanical neck pain	Acupuncture	Physiotherapy	IG:15 CG:15	≥ 3 months	10 weeks, 6 months after completion of treatment	VAS, NDI	NR
**MacPherson 2015, UK** [28]	Chronic neck pain	Acupuncture	Usual care	IG:173 CG:172	≥ 3 months	1, 3, 12 months after completion of treatment	NPQ	14%/5%
**De 2017, Belgium** [30]	Chronic mechanical neck pain	Dry needling	Manual Pressure Technique	IG:22 CG:20	≥ 3 months	3 months after completion of treatment	NRS, NDI	NR
**Nejati 2021, Iran** [33]	Chronic neck pain	Acupuncture	Exercise	IG:34 CG:34	≥ 3 months	1, 6 months after completion of treatment	NDI	NR
**Valiente 2021, Spain** [34]	Chronic mechanical neck pain	Dry needling	Usual care	IG:21 CG:20	≥ 3 months	1, 3 months after completion of treatment	VAS, NDI	NR
**Lin 2017, China** [36]	Cervical spondylosis	Warm needle moxibustion + Traction therapy	Traction therapy	IG:35 CG:35	IG:18.03 ± 18.97 CG:16.86 ± 25.35	3 months after completion of treatment	NRS	NR
**Huang 2019, China** [37]	Chronic mechanical neck pain	Press needle + TENS	TENS	IG:40 CG:40	≥ 6 months	2 weeks, 1, and 6 months after completion of treatment	VAS, NDI	NR
**Tang 2022, China** [38]	Chronic mechanical neck pain	Acupuncture + Massage	Massage	IG:40 CG:40	IG:5.8 ± 3.8 CG:5.6 ± 4.2 (months)	1 week, 3 months after completion of treatment	VAS, MPQ, NDI	NR
**Ma 2010, China** [26]	Chronic mechanical neck pain	Acupuncture + Self neck—stretching exercises	Self-neck—stretching exercises	IG:15 CG:13	≥ 6 months	2 weeks, 3 months after completion of treatment	VAS	74%/ NR
**Cerezo 2016, Spain** [29]	Chronic mechanical neck pain	Dry needling + Stretching	Stretching	IG:64 CG:64	≥ 6 months	15,30,90, 180 days after completion of treatment	VAS, NDI	NR
**Stieven 2020, Brazil** [32]	Chronic neck pain	Dry needling + Physical therapy	Physical Therapy	IG:58 CG:58	≥ 3 months	1, 3, 6 months after completion of treatment	NDI	13%/10.3%

*IG* intervention group, *CG* control group, *TENS* Transcutaneous electrical nerve stimulation, *NPQ* Neck Pain Questionnaire, *NRS* numerical rating scale, *VAS* Visual analog scale, *NDI* neck dysfunction index, *MPQ* McGill Pain Questionnaire, *SF-36* 36-Item Short Form Health Survey, *NR* not reported

### Risk of Bias Assessment

**Fig. 2:**
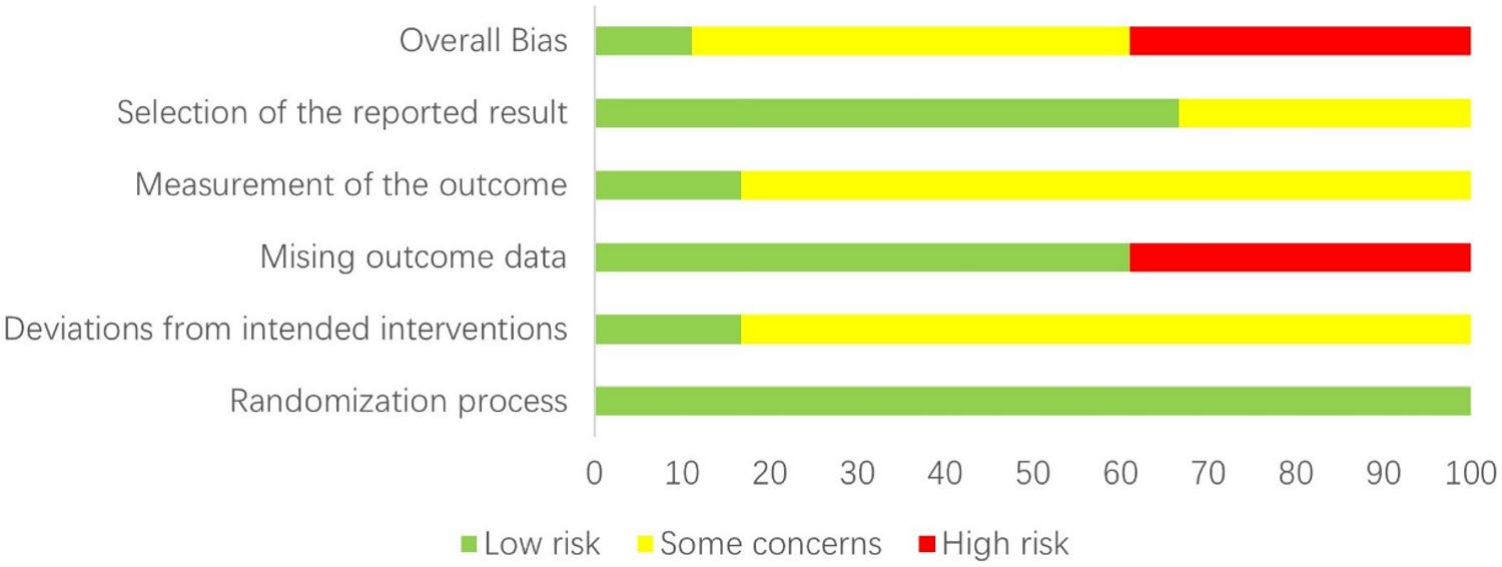
Risk of bias graph

**Fig. 3:**
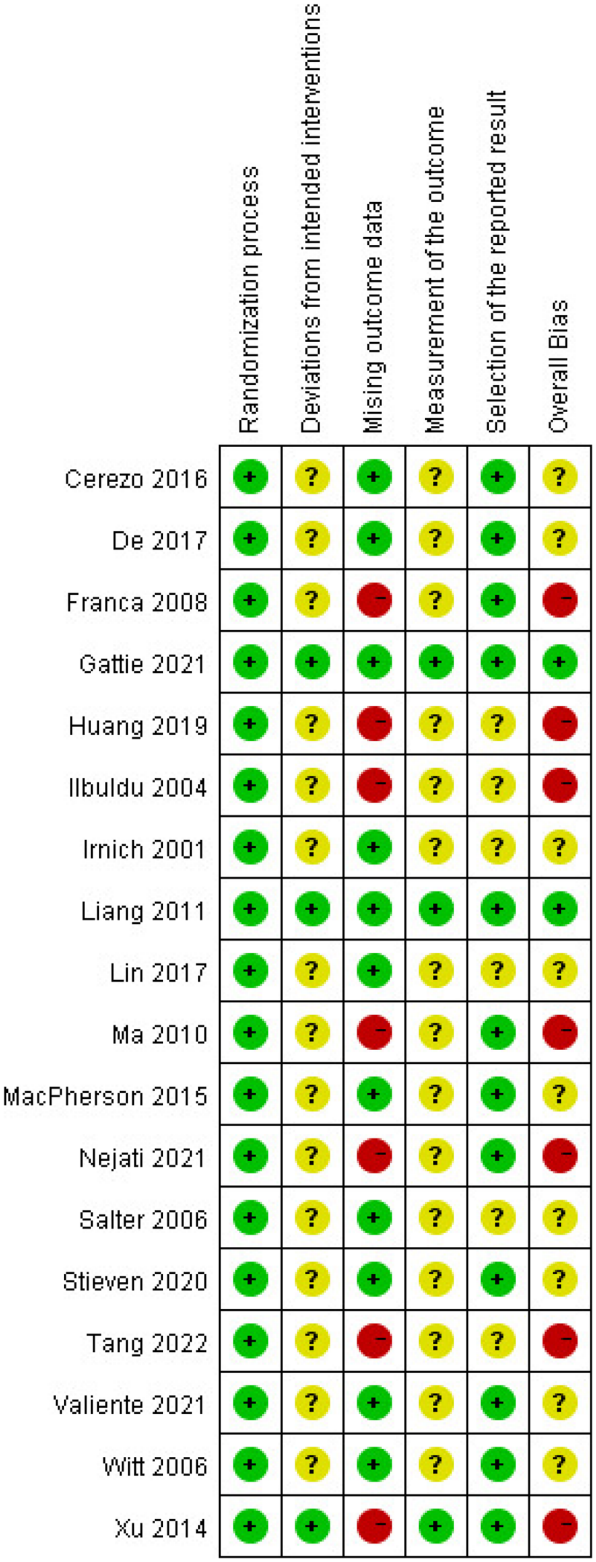
Risk of Bias of included studies

In our meta-analysis of 18 studies, rigorous randomization resulted in a low risk in this domain. However, 15 studies [21–26, 28–30, 32–34, 36–38] lacked detailed descriptions of intervention deviation and adequate blinding, posing a latent risk of bias. Regarding missing outcome data, 11 studies [21, 22, 24, 27–32, 34, 36] meticulously reported participant withdrawals and final analysis inclusions, substantiating a low bias risk, while seven studies [23, 25, 26, 33, 35, 37] failed to provide a study flowchart, thereby obscuring the precise number of patients lost to follow-up and thus leading to a high risk of bias. Six studies [22–24, 36–38] showed unclear selective reporting bias owing to the lack of protocol registration, whereas the remaining 12 [21, 25–35] had a low bias risk in this domain. Overall and individual trial bias risks are both presented in Figs. 2 and 3.

### Acupuncture Versus Sham Acupuncture

#### Pain Intensity

Two studies evaluated the prolonged effects of acupuncture versus sham acupuncture on CNP by assessing the VAS scores at 3, 6, and 12 months post-treatment. Liang et al. [27] reported no statistically significant difference at 3 months (MD: − 0.12; 95% CI − 0.06 to 0.36; p = 0.63) (Supplementary Fig. 1). Similarly, Gattie et al. [31] observed no statistically significant difference at 6 months (MD: 0.01; 95% CI − 1.16 to 1.18; p = 0.99) (Supplementary Fig. 2), a trend that continued at 12 months (MD: − 0.42; 95% CI − 1.55 to 0.71; p = 0.47) (Supplementary Fig. 3).

#### Disability

**Fig. 4:**
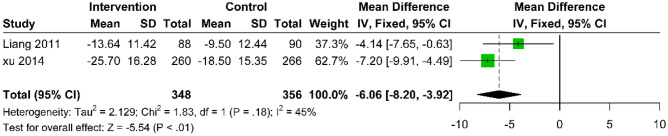
Forest plot of the mean difference in change of NPQ scores between acupuncture and sham acupuncture at the 3-month follow-up after intervention, compared to baseline, for CNP

Three studies compared acupuncture with sham acupuncture in functional improvements using the NDI and NPQ. At 6 months post-treatment, Gattie et al. [31] observed no statistically significant difference in NDI scores (MD: 2.40; 95% CI − 5.46 to 10.26; p = 0.55) (Supplementary Fig. 4). At 12 months, the difference in NDI scores remained insignificant (MD: − 0.11; 95% CI − 7.69 to 7.47; p = 0.98) (Supplementary Fig. 5). However, significant functional benefits were observed at 3 months post-treatment for NPQ. The synthesized data from Liang et al. [27] and Xu et al. [35] showed a notable improvement (MD: − 6.06; 95% CI − 8.20 to − 3.92; p < 0.01; I^2^ = 45%) (Fig. 4). Nevertheless, this improvement did not reach the minimal clinically important difference (MCID) criterion, defined as a 25% reduction in score from baseline [39].

#### Quality of Life

The study by Liang et al. [27] found that observations 3 months post-treatment revealed that acupuncture did not exhibit statistically significant improvements over sham acupuncture in both mental component summary (MCS) scores (MD: 5.36; 95% CI: − 1.53 to 12.25; p = 0.13) (Supplementary Fig. 6) and physical component summary (PCS) scores (MD: 1.02; 95% CI: − 6.20 to 8.24; p = 0.78) (Supplementary Fig. 7).

### Acupuncture Versus No-Treatment Control

The study by Witt et al. [21] investigated the effect of acupuncture on the quality of life compared with no-treatment using the SF-36. At 3 months post-treatment, acupuncture provided a statistically significant improvement in MCS (MD: 3.20; 95% CI 1.30 to 5.10; p = 0.001) (Supplementary Fig. 8), meeting the MCID threshold of 2.5 [40]. However, this improvement diminished at 6 months post-treatment (MD: 0.90; 95% CI 0.36 to 1.44; p = 0.001) (Supplementary Fig. 9). The PCS scores initially improved significantly at 3 months post-treatment (MD: 4.60; 95% CI 1.86 to 7.34; p = 0.001) (Supplementary Fig. 10), exceeding the MCID of 2.6 [41], but the effect size decreased at 6 months post-treatment (MD: 0.60; 95% CI 0.24 to 0.96; p = 0.001) (Supplementary Fig. 11). The study by Witt et al. did not report on pain intensity or function.

### Acupuncture Versus Active Control

#### Pain Intensity

**Fig. 5:**
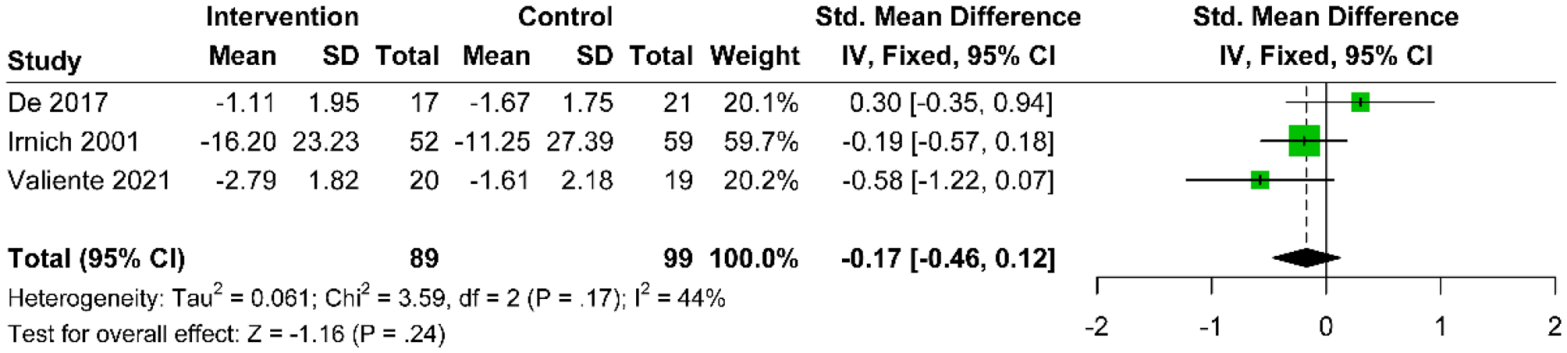
Forest plot comparing the standardized mean difference in pain score changes between acupuncture and active control at the 3-month follow-up after intervention, relative to baseline, for CNP

**Fig. 6:**
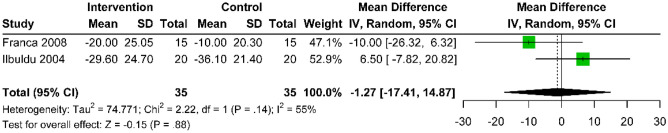
Forest plot comparing the mean difference in pain score changes between acupuncture and active control at the 3-month follow-up after intervention, relative to baseline, for CNP

Five studies [22, 23, 25, 30, 34] compared the efficacy of acupuncture with active controls in the management of pain intensity, as measured by the VAS or NRS scoring systems. De et al., Irnich et al., and Valiente et al. compared acupuncture with active treatment in VAS or NRS scores three months after treatment (MD: − 0.17; 95% CI − 0.46 to 12; p = 0.24; I^2^ = 44%) (Fig. 5), while the studies by Franca et al. and Ilbuldu et al. made a similar comparison at six months (MD: − 1.27; 95% CI − 17.41 to 14.87; p = 0.88; I^2^ = 55%) (Fig. 6). No statistically significant differences were observed in either time frame.

#### Disability

**Fig. 7:**
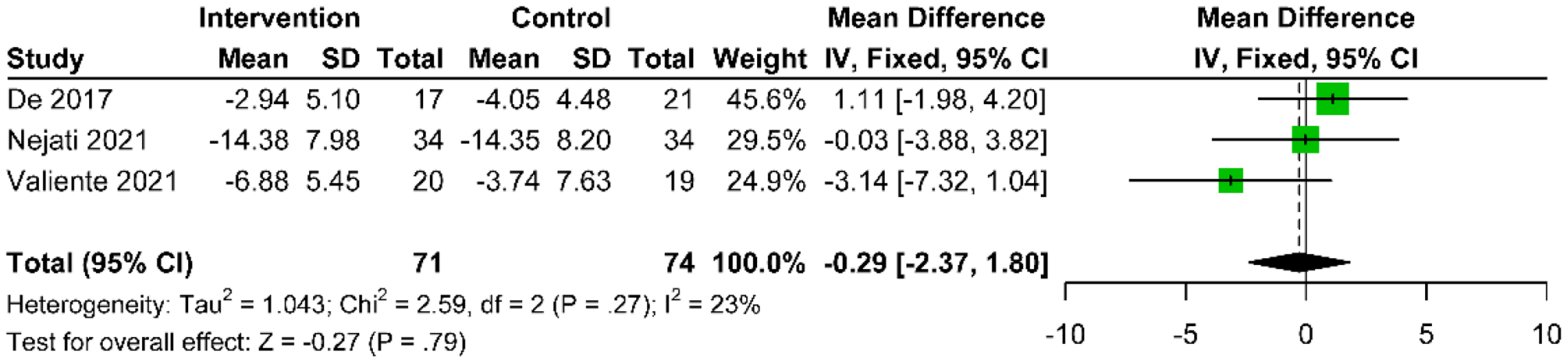
Forest plot of the mean difference in change of NDI scores between acupuncture and active control at the 3-month follow-up after intervention, compared to baseline, for CNP

**Fig. 8:**
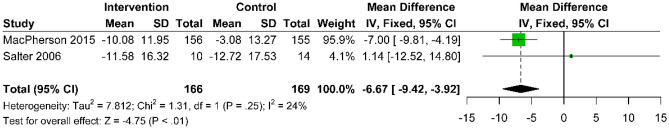
Forest plot of the mean difference in change of NPQ scores between acupuncture and active control at the 3-month follow-up after intervention, compared to baseline, for CNP

Six studies [24, 25, 28, 30, 33, 34] analyzed the long-term efficacy of acupuncture versus active control using the NDI and NPQ to measure disability and functional improvement. At 3 months post-treatment, NDI showed no significant difference (MD: − 0.29; 95% CI − 2.37 to 1.80; p = 0.79; I^2^ = 23%) (Fig. 7), but at 6 months, a significant difference was observed (MD: − 9.00; 95% CI − 14.06 to − 3.94; p = 0.0005) (Supplementary Fig. 12), meeting the MCID of 3 points [41]. NPQ results indicated significant improvement at 3 months post-treatment (MD: − 6.67; 95% CI − 9.42 to − 3.92; p < 0.01; I^2^ = 24%) (Fig. 8), persisting at 6 (MD: − 6.33; 95% CI − 9.22 to − 3.44; p < 0.0001) (Supplementary Fig. 13) and 12 months (MD: − 4.75; 95% CI − 7.86 to − 1.64; p = 0.003) (Supplementary Fig. 14).

### Acupuncture with Active Control Versus Active Control (add-on)

#### Pain Intensity

**Fig. 9:**
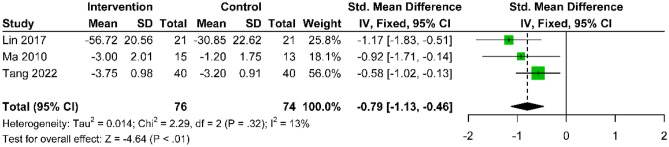
Forest plot comparing the standardized mean difference in pain score changes between acupuncture with active control and active control at the 3-month follow-up after intervention, relative to baseline, for CNP

**Fig. 10:**
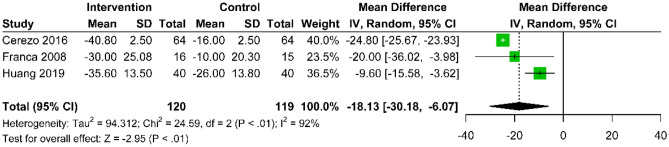
Forest plot comparing the mean difference in pain score changes between acupuncture with active control and active control at the 6-month follow-up after intervention, relative to baseline, for CNP

Six studies rigorously evaluated the long-term effectiveness of combining acupuncture with active control interventions versus active control alone for treating CNP. These studies employed various pain assessment tools, including the VAS in five studies [25, 26, 29, 37, 38] to measure pain intensity, the NRS in one study [36], and the MPQ for a comprehensive evaluation of pain in another [38]. At the three-month post-treatment mark, a fixed-effects model analysis of VAS (0–10) and NRS scores (0–100) showed a standardized mean difference (SMD) favoring acupuncture combined with active control over active control alone (SMD: − 0.79; 95% CI − 1.13 to − 0.46; p < 0.01; I^2^ = 13%) (Fig. 9). At the six-month post-treatment mark, a random-effects model analysis of VAS scores (0–100) indicated that acupuncture with active control maintained a benefit over active control alone (MD: − 18.13; 95% CI − 30.18 to − 6.07; p < 0.01), although with a substantial heterogeneity (I^2^ = 92%) (Fig. 10). The MPQ reported a mean difference (MD: − 1.03; 95% CI − 2.38 to 0.32; p = 0.13) (Supplementary Fig. 15), meeting the MCID of 1 for MPQ, though it did not reach statistical significance.

#### Disability

**Fig. 11:**
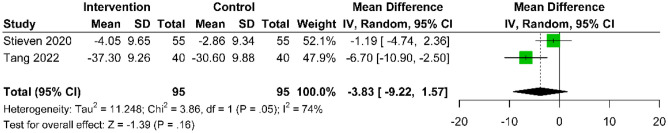
Forest plot of the mean difference in change of NDI scores between acupuncture with active control and active control at the 3-month follow-up after intervention, compared to baseline, for CNP

**Fig. 12:**
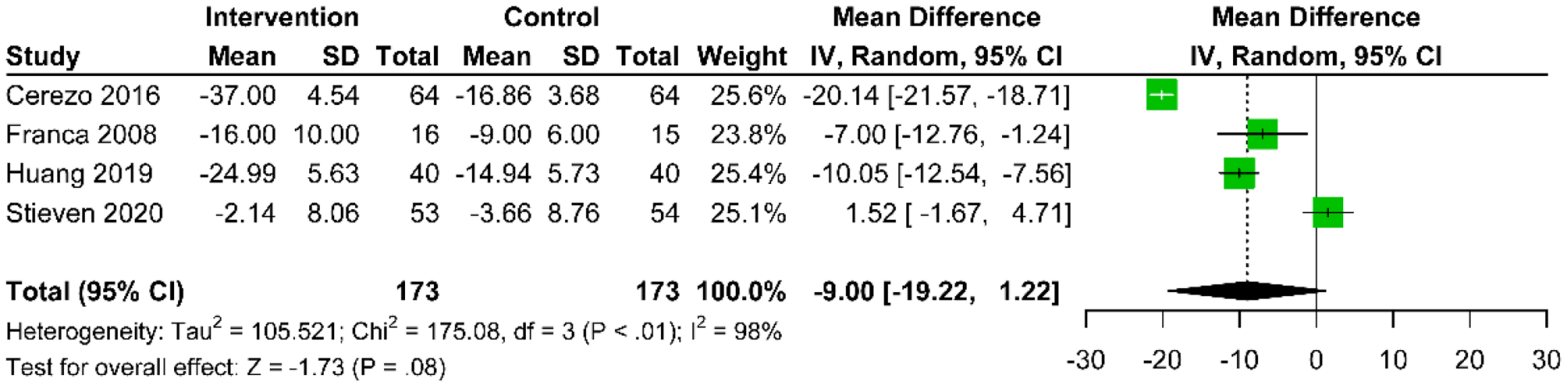
Forest plot of the mean difference in change of NDI scores between acupuncture with active control and active control at the 6-month follow-up after intervention, compared to baseline, for CNP

Five studies compared the efficacy of acupuncture combined with active treatment with active treatment alone. Two studies [32, 38 ] reported outcomes at 3 months post-treatment (MD: − 3.83; 95% CI − 9.22 to 1.57; p = 0.16; I^2^ = 74%) (Fig. 11), and four studies [25, 29, 32, 37] provided data at 6 months (MD: − 9.00; 95% CI − 19.22 to 1.22; p = 0.08; I^2^ = 98%) (Fig. 12), with no statistical significance.

### Safety Assessment

Among the 18 included studies, eight did not report on adverse events, while 10 noted mild adverse events with no severe cases reported. The incidence rate of adverse events in the acupuncture group was 8.5% compared to 3% in the sham acupuncture group, 6.4% in the active treatment group, and 13.8% in the combined acupuncture and active treatment groups. The minor adverse effects associated with acupuncture reported in most studies [25, 29, 31, 32, 34] included minor local bleeding or hematoma after acupuncture and needling pain, with a likelihood of occurrence at 23.7%. Studies by Salter et al. [24] and Stieven et al. [32] indicate a 13.2% chance of transient dizziness, 5.9% of fatigue, and 20.6% of worsening symptoms associated with acupuncture. Liang et al. [27] reported that three patients in the acupuncture group fainted during treatment, and the symptoms were relieved entirely after lying down and drinking hot water.

## Discussion

The objective of this review was to evaluate the sustained effects of acupuncture on CNP. CNP characterized by its prolonged course and tendency for recurrence, necessitated extended follow-up periods to adequately assess the sustained effects of acupuncture. The majority of included studies in our analysis opted for follow-up time points of one month (55%), three months (78%), six months (39%), and 12 months (11%) after acupuncture treatment. In evaluating persistent effects, most studies favored follow-up periods exceeding three months [43, 44••]. Consequently, our meta-analysis primarily focused on the outcomes at 3, 6 and 12 months post-treatment.

Compared to sham acupuncture, acupuncture did not demonstrate sustained effects in alleviating pain or improving the quality of life. This finding suggests two possible interpretations: first, acupuncture may not provide a lasting impact in reducing the severity of CNP and enhancing the quality of life; second, sham acupuncture interventions could exhibit therapeutic benefits, potentially due to the placebo effect influenced by patient expectations and beliefs, impacting treatment outcomes [44••]. However, in terms of functional improvements, acupuncture showed certain advantages over sham acupuncture, especially in terms of NPQ scores at the three-month follow-up. This might indicate that the effect of acupuncture on sustained functional recovery surpasses those of psychological factors. Pain and quality of life ratings are susceptible to influences from emotional states, environmental factors, and individual expectations. In contrast, functional status assessments (such as the NPQ scale) may reflect objective physical abilities and the ability to perform daily activities more accurately. Therefore, changes in these domains may not necessarily align perfectly. In the follow-up comparisons between the acupuncture and active control groups, acupuncture did not show significant statistical or clinical differences in pain intensity compared to the active controls. This suggests that its effectiveness in the ongoing control of CNP may be comparable to conventional medication or non-acupuncture physical therapy. However, regarding functional impairment, acupuncture demonstrated a significant improvement at the six-month mark (NDI score), reaching MCID. Furthermore, statistically significant improvements in NPQ scores were observed at 3, 6, and 12 months, indicating that acupuncture may be superior in enhancing functional status and reducing disability than active control. In studies comparing acupuncture combined with active control to active control alone, the combination of acupuncture and active control was more effective in alleviating pain, suggesting that acupuncture as an adjunct therapy may significantly enhance the therapeutic efficacy of conventional treatments. Significant heterogeneity was observed in studies comparing acupuncture with active controls, which may be attributed to variations in the types and intensities of active controls used across studies. Only one study compared acupuncture with a no-treatment group, suggesting that acupuncture might have sustained the effects of acupuncture in improving quality of life.

Despite the mechanisms underlying the efficacy of acupuncture not being fully elucidated, acupuncture stimulation is widely believed to trigger inherent pain control mechanisms in the body, thereby producing analgesic effects [45]. Particularly, neuroplasticity provides a rational explanation for the long-term analgesic effects of acupuncture. For instance, in rat models, low-frequency (2 Hz) electroacupuncture has been shown to induce long-term depression (LTD) in C fibers, leading to sustained pain alleviation [46].

Our findings align with those reported in the review conducted by Yuan et al. [47], which included 17 studies with 1,434 participants. The aggregated evidence from these studies was rated as high-quality. The authors synthesized results from multiple follow-up intervals, revealing that the positive effects of acupuncture on functional impairment were sustained for more than three months. Furthermore, pain reduction was not significantly different compared to sham acupuncture during the follow-up phase. Our investigation extends the follow-up period beyond their scope, appraising the persistent effects at 6 and 12 months post-treatment and evaluating the contribution of acupuncture to enhancing the quality of life.

Acupuncture improves functional status with sustained effects, and as an adjunctive therapy, acupuncture provides sustained relief from pain, Thus, its advantages extend beyond the treatment duration, providing more than just temporary relief of symptoms. This finding is crucial for clinical practice as it addresses a major concern for both patients and healthcare providers: consistent relief can lead to less frequent treatments, thus facilitating more effective CNP management. Another notable aspect of this investigation lies in the outstanding safety profile exhibited by acupuncture, which underscores its potential for wider clinical adoption. Our study has certain limitations. First, there is the predominantly moderate-to-low quality of most of the evidence included. This limitation arises from several factors: a relatively limited scope of literature, with a few outcome indicators reported in only a single source; varied definitions, diagnostic criteria, and treatment methods for CNP across different studies that may have impacted the results; and incomplete assessment of the adverse effects of acupuncture in studies. Furthermore, the articles in our analysis did not report whether patients received any other treatments during the follow-up period, necessitating a cautious interpretation of our results, as any additional treatments could have influenced the outcomes observed post-treatment. We believe that future studies on acupuncture should emphasize its prolonged effects, with follow-ups exceeding three months while ensuring a high-quality design and adequate sample sizes for reliable results.

## Conclusion

Acupuncture, as an adjunct therapy, may provide post-treatment pain relief for at least three months among patients with CNP, although its effectiveness is not superior to that of sham acupuncture. The benefits of acupuncture in improving functional impairments extend beyond three months. Despite the necessity for further investigation requiring increased sample sizes, standardized procedures, and meticulous designs, the enduring therapeutic effect of acupuncture and its favorable safety profile, as evidenced by existing research, suggests that it may be a complementary approach for managing CNP.

## Supplementary Information

Below is the link to the electronic supplementary material.

Supplementary file1 (PDF 185 KB)

## Data Availability

All data generated in this review are available upon request.
