# Supplementary material for: Durable Effect of Acupuncture for Chronic Neck Pain: A Systematic Review and Meta-Analysis
Source: Curr Pain Headache Rep. 2024 Jun 10;28(9):957–69. doi: 10.1007/s11916-024-01267-x (PMC11416387; doi:10.1007/s11916-024-01267-x)
Supplement: Supplementary file 1 — Supplementary file1 (PDF 185 KB) [file 11916_2024_1267_MOESM1_ESM.pdf]

Supplementary Information

Durable effect of acupuncture for chronic neck pain: a systematic review and meta-analysis

Current Pain and Headache Reports

Jiufei Fang[1¶], Hangyu Shi[1,2¶], Weiming Wang[1], He Chen[1], Min Yang[1], Shuai Gao[1], Hao Yao[1,2], Lili Zhu[1], Yan Yan[1], Zhishun Liu[1*]

[1]Department of Acupuncture, Guang'anmen Hospital, China Academy of Chinese Medical Sciences, Beijing, China; [2]Graduate College, Beijing University of Chinese Medicine, Beijing, China.

¶These authors contributed equally to this work

*Correspondence: Zhishun Liu

Department of Acupuncture, Guang'anmen Hospital, China Academy of Chinese Medical Sciences, No.5 Beixiange St., Xicheng District, Beijing, China,100053

Tel: +86 13651016313

Email: zhishunjournal@163.com

Search strategy and results

Table 2 PubMed search strategies

**PubMed**	
(("Acupuncture"[Mesh]) OR ((((((((((((acupuncture-moxibustion[Title/Abstract]) OR (acupuncture therapy[Title/Abstract])) OR (needle moxibustion[Title/Abstract])) OR (acupuncture treatment[Title/Abstract])) OR (electroacupuncture[Title/Abstract])) OR (Manual acupuncture[Title/Abstract])) OR (moxi-acupuncture[Title/Abstract])) OR (Needling[Title/Abstract])) OR (acupressure[Title/Abstract])) OR (Electric acupuncture[Title/Abstract])) OR (acupuncture [Title/Abstract])) OR (dry needling[Title/Abstract]))) AND (("Neck Pain"[Mesh]) OR ((((((((((((((cervical pain[Title/Abstract]) OR (painful neck[Title/Abstract])) OR (neck disorder[Title/Abstract])) OR (cervicodynia [Title/Abstract])) OR (cervical spondylosis[Title/Abstract])) OR (cervical radiculopathy[Title/Abstract])) OR (cervical disc[Title/Abstract])) OR (herniation[Title/Abstract])) OR (myofascial pain syndrome[Title/Abstract])) OR (trachelodynia[Title/Abstract])) OR (brachelodynia[Title/Abstract])) OR (trachelagra[Title/Abstract])) OR (chronic spinal pain[Title/Abstract])))	774

Table 3 Cochrane Library search strategies

**Cochrane Library**	
#1 MeSH descriptor:[Neck Pain]explode all trees	11109
#2 (cervical pain):ti,ab,kw OR(painful neck):ti,ab,kw OR (neck disorder):ti,ab,kw OR(cervicodynia):ti,ab,kw OR(cervical spondylosis):ti,ab,kw	9204
#3 (cervical radiculopathy):ti,ab,kw OR(cervical disc):ti,ab,kw OR (herniation):ti,ab,kw OR(myofascial pain syndrome):ti,ab,kw OR (trachelodynia):ti,ab,kw	4827
#4 (brachelodynia):ti,ab,kw OR(trachelagra):ti,ab,kw OR(chronic spinal pain):ti,ab,kw	3270
#5 MeSH descriptor:[Acupuncture]explode all trees	21477
#6 (acupuncture-moxibustion):ti,ab,kw OR(acupuncture therapy):ti,ab,kw OR(needle moxibustion):ti,ab,kw OR (acupuncture treatment):ti,ab,kw AND (electroacupuncture):ti,ab,kw	11348
#7 (acupunture):ti,ab,kw OR(dry needling):ti,ab,kw	1348
#8 #1 or #2 or #3 or #4	22198

#9 #5or#6or#7	22414
#10 #8 and #9	1674

Table 4 EMBASE search strategies

**EMBASE**	
#1 neck pain'/exp	32191
#2 'cervical pain':ab,ti OR 'painful neck':ab,ti OR 'neck disorder':ab,ti OR cervicodynia:ab,ti OR 'cervical spondylosis':ab,ti OR 'cervical radiculopathy':ab,ti OR 'cervical disc':ab,ti OR herniation:ab,ti OR 'myofascial pain syndrome':ab,ti OR trachelodynia:ab,ti OR brachelodynia:ab,ti OR trachelagra:ab,ti OR 'chronic spinal pain':ab,ti	41169
#3 #1OR#2	136748
#4 acupuncture'/exp	67461
#5 #3 AND #4	1629

Table 5 CNKI search strategies

**CNKI**	
(((((((((("Neck Pain" in Topic or "Neck Pain" in Title) OR ("Chronic Neck Pain" in Topic or "Chronic Neck Pain" in Title)) OR ("Xiangjiang" in Topic or "Xiangjiang" in Title)) OR ("Neck Pain" in Topic or "Neck Pain" in Title)) OR ("Xiangbi" in Topic or "Xiangbi" in Title)) OR ("Neck and Shoulder Pain" in Topic or "Neck and Shoulder Pain" in Title)) OR ("Cervical Spondylosis" in Topic or "Cervical Spondylosis" in Title)) AND (((((("Acupuncture" in Topic or "Acupuncture" in Title) OR ("Moxibustion" in Topic or "Moxibustion" in Title)) OR ("Electroacupuncture" in Topic or "Electroacupuncture" in Title)))) AND (((((("Randomized" in Abstract) OR ("Blind Study" in Abstract)) OR ("Double Blind" in Abstract)) OR ("Single Blind" in Abstract)) OR ("Controlled" in Abstract))))	1311

Table 6 WANFANG search strategies

**WANFANG**	
(Title:(Neck Pain) or Title:(Chronic Neck Pain) or Title:(Xiangjiang) or Title:(Neck and Shoulder Pain) or Title:(Xiangbi) or Title:(Neck and Shoulder Pain) or Title:(Cervical	1488

Spondylosis)) and (Title:(Acupuncture) or Title:(Moxibustion) or Title:(Electroacupuncture)) and (Title:(Randomized) or Title:(Blind Study) or Title:(Double Blind) or Title:(Single Blind) or Title:(Controlled))

Table 7 VIP Database search strategies

**VIP Database**
((((((((((((((((((((((Title or Keywords = Neck Pain OR Title or Keywords = Chronic Neck Pain) OR Title or Keywords = Xiangjiang) OR Title or Keywords = Neck and Shoulder Pain) OR Title or Keywords = Xiangbi) OR Title or Keywords = Neck and Shoulder Pain) OR Title or Keywords = Cervical Spondylosis) AND ((Title or Keywords = Acupuncture OR Title or Keywords = Moxibustion) OR Title or Keywords = Electroacupuncture))))))))) AND ((((Abstract = Randomized OR Abstract = Blind Study) OR Abstract = Double Blind) OR Abstract = Single Blind) OR Abstract = Controlled))))))))) AND (Publication Year:[1900 TO 2022])

763

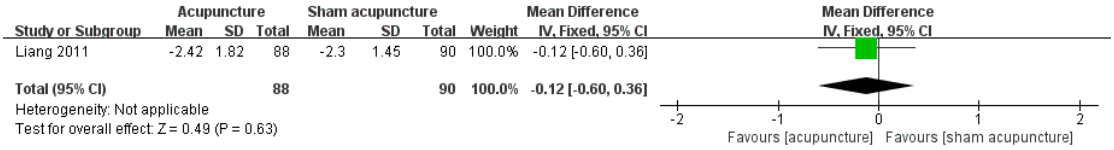

Fig. 1 Forest plot of mean difference in change of VAS score between acupuncture and sham acupuncture at the 3-month follow-up after intervention, compared to baseline, for CNP

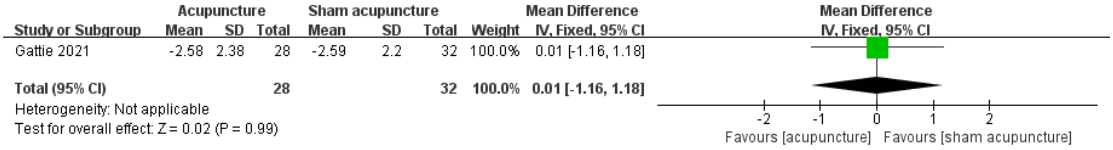

Fig. 2 Forest plot of mean difference in change of VAS score between acupuncture and sham acupuncture at the 6-month follow-up after intervention, compared to baseline, for CNP

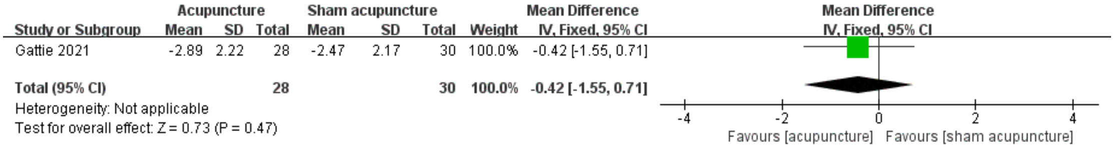

Fig. 3 Forest plot of mean difference in change of VAS score between acupuncture and sham

acupuncture at the 12-month follow-up after intervention, compared to baseline, for CNP

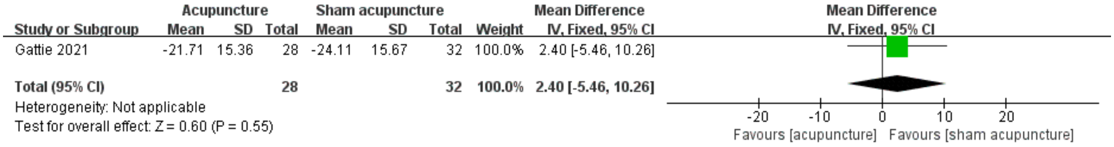

Fig. 4 Forest plot of mean difference in change of NDI score between acupuncture and sham acupuncture at the 6-month follow-up after intervention, compared to baseline, for CNP

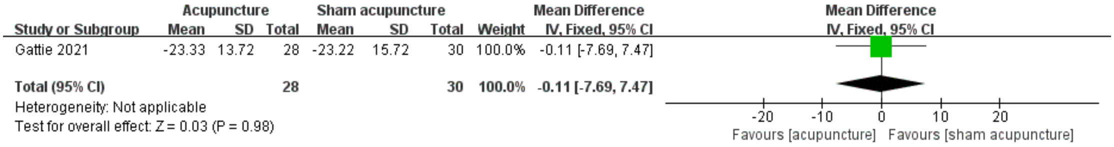

Fig. 5 Forest plot of mean difference in change of NDI score between acupuncture and sham acupuncture at the 12-month follow-up after intervention, compared to baseline, for CNP

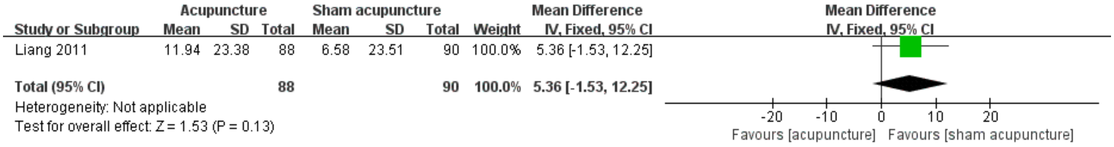

Fig. 6 Forest plot of mean difference in change of MCS score between acupuncture and sham acupuncture at the 3-month follow-up after intervention, compared to baseline, for CNP

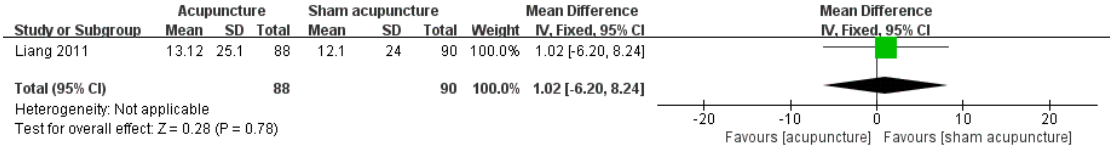

Fig. 7 Forest plot of mean difference in change of PCS score between acupuncture and sham acupuncture at the 3-month follow-up after intervention, compared to baseline, for CNP

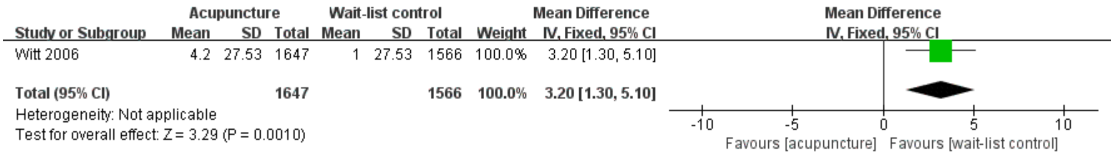

Fig. 8 Forest plot of mean difference in change of MCS score between acupuncture and no-treatment at the 3-month follow-up after intervention, compared to baseline, for CNP

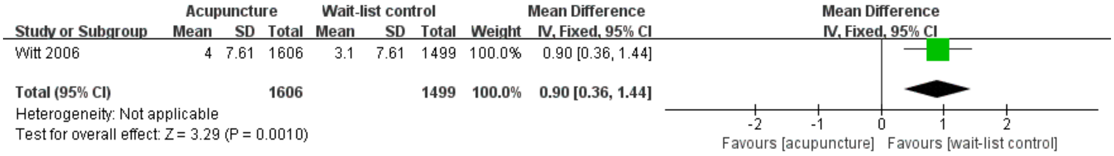

Fig. 9 Forest plot of mean difference in change of MCS score between acupuncture and no-treatment at the 6-month follow-up after intervention, compared to baseline, for CNP

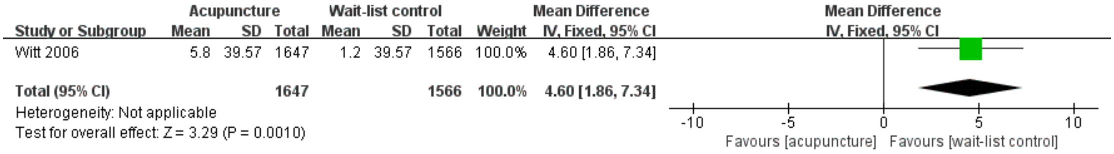

Fig. 10 Forest plot of mean difference in change of PCS score between acupuncture and no-treatment at the 3-month follow-up after intervention, compared to baseline, for CNP

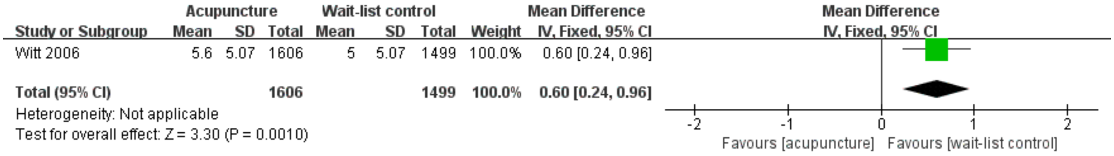

Fig. 11 Forest plot of mean difference in change of PCS score between acupuncture and no-treatment at the 6-month follow-up after intervention, compared to baseline, for CNP

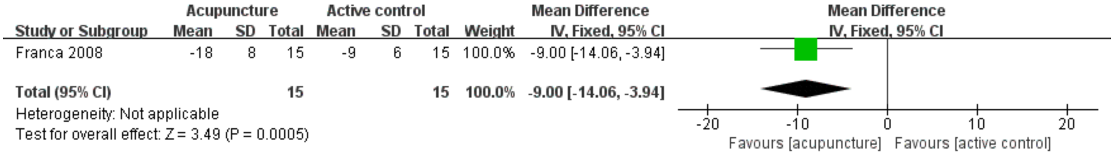

Fig. 12 Forest plot of mean difference in change of NDI score between acupuncture and active control at the 6-month follow-up after intervention, compared to baseline, for CNP

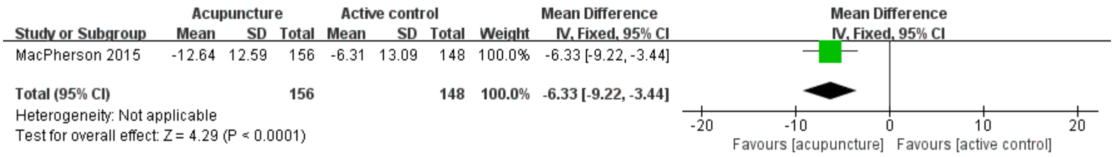

Fig. 13 Forest plot of mean difference in change of NPQ score between acupuncture and active control at the 6-month follow-up after intervention, compared to baseline, for CNP

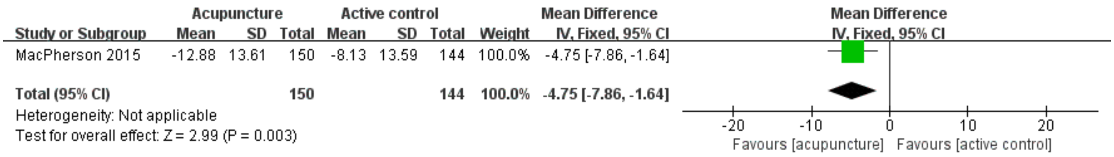

Fig. 14 Forest plot of mean difference in change of NPQ score between acupuncture and active control at the 12-month follow-up after intervention, compared to baseline, for CNP

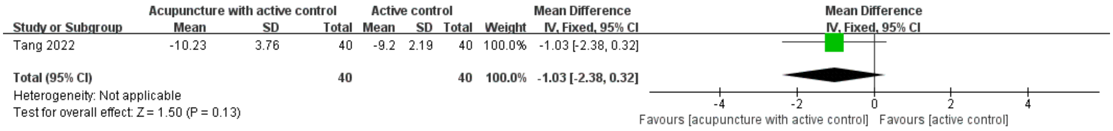

Fig. 15 Forest plot of mean difference in change of MPQ score between acupuncture combined with active control and active control alone at the 3-month follow-up after intervention, compared to baseline, for CNP
